# Addenda to Paper on Opium Habit

**Published:** 1915-09

**Authors:** 


### ADDENDA TO PAPER ON OPIUM HABIT

It seems inconsistent to announce the permanent removal in a few days or weeks of the craving for habit-forming drugs. We must remember that this craving, dependent on perverted, deep-rooted habits in mind and body, has become so dominant that its satisfaction is, in a sense, a biologic necessity. In reality the craving for a drug such as morphine is rarely obliterated by a short-time treatment. In most cases the appetite is but an abeyance so long as the patient is in a condition such that under given circumstances the craving will again spring into activity. There has been produced in the habitue a lowering of resistance for withstanding either in the mental or physical realm. Flight from pain or the possibility of pain, has become an obsession. In circumstances, therefore, in which distress, or the expectation of it arises, the individual is often helpless; in his panic of fear he rushes instinctively to that which his memory informs him will give him relief—his Drug. He is under the yoke of his associative memory processes, and in such times of stress, his craving dominates him, body and soul.

We who advocate specific treatment, such as with Hyoscine, warn against the first dose. It is rather our business to protect the patient for a sufficient length of time from conditions which may create what may SEEM TO HIM A NECESSITY for the first dose.

Permanent abstinence, in the majority of cases, will come only when an enduring incentive has been established, which is a stronger urge than the impulse to find a nepenthe in drugs.

Candler Building, Atlanta, Ga.

